# Validation of Screening Tools for Predicting the Risk of Functional Decline in Hospitalized Elderly Patients

**DOI:** 10.3390/ijerph19116685

**Published:** 2022-05-30

**Authors:** Mei-Chun Wang, Wen-Chun Liao, Kwo-Chen Lee, Shu-Hua Lu, Yun-Ping Lin

**Affiliations:** 1 Department of Nursing, Kuang Tien General Hospital, Taichung 437021, Taiwan; meis567@hotmail.com.tw; 2 School of Nursing, China Medical University, Taichung 406040, Taiwan; wcl@mail.cmu.edu.tw (W.-C.L.); rubylee@mail.cmu.edu.tw (K.-C.L.); 3 Department of Nursing, China Medical University Hospital, Taichung 404332, Taiwan

**Keywords:** hospitalized elderly patients, functional decline, screening tools, validity

## Abstract

Background: Functional decline and increased dependence on others are common health issues among hospitalized elderly patients. However, a well-validated screening tool for predicting functional decline in elderly patients is still lacking. The current study therefore aimed to evaluate and compare the diagnostic accuracy of the Identification of Seniors at Risk—Hospitalized Patients (ISAR-HP), Variable Indicative of Placement Risk (VIP), and Score Hospitalier d’ Evaluation du Risque de Perte d’Autonomie (SHERPA) in predicting functional decline 30 days after discharge in older patients admitted to an acute hospital ward. Methods: A prospective, longitudinal study was conducted in 197 elderly inpatients at the internal medicine ward of a teaching hospital in central Taiwan. Data were collected twice, first within 48 h after hospitalization and second via a telephone interview 30 days after hospital discharge. Variables included demographic data, Barthel Index of activities of daily living (ADL), and screening instruments. The Barthel Index was used to measure functional disability. Functional decline was defined as a decline of at least five points on the Barthel Index 30 days after discharge compared to that at pre-admission. Results: Patients had a mean age of 77.7 years, with 55.7% being female. Functional decline was observed in 39.1% of all patients. The best cutoff point, sensitivity, specificity, and area under the receiver operating characteristic curve were 2.5, 96.1%, 52.5%, and 0.751 for ISAR-HP; 1.5, 83.1%, 62.5%, and 0.761 for VIP; and 4.75, 89.6%, 54.2%, and 0.758 for SHERPA, respectively. Conclusions: All three instruments showed moderate diagnostic accuracy as indicated by their best cutoff points. Therefore, the results presented herein can guide health care professionals in selecting the appropriate assessment tool for predicting functional decline among hospitalized elderly patients in a clinical setting.

## 1. Introduction

The elderly population continues to grow at an alarming pace worldwide. In line with this, approximately 30–60% of patients aged 65 and over were found to experience functional decline during hospitalization [1,2,3], resulting in decreased autonomy, increased dependence, rise in medical cost and family burden, prolonged hospitalization, growth in readmission rate, and long-term institutionalization in a care facility [4,5]. 

However, early detection of functional decline benefits from early intervention to prevent and manage frailty. Exercise intervention has proved to reverse functional decline and improve cognitive function among acutely hospitalized very elderly patients [6]. An interdisciplinary team approach, such as the Hospital Elder Life Program (HELP) [7], also has a significant impact on reducing the risk for delirium and falls, with a trend for a decrease in the length of stay and preventing institutionalization [8]. 

Functional decline is usually defined as a decline in activities of daily living (ADLs), an indicator of functional independence in self-care activities, and/or instrumental activities of daily living (IADLs), an indicator of an individual’s ability to perform independent living skills such as shopping, using a telephone, doing laundry, preparing meals, housekeeping, taking medications, using public transportation, and handling finances [9]. Functional decline has also been used to refer to a wide range of functional status, such as tasks performed by a person necessary to live independently in the community, including physical, psychological, spiritual, social, intellectual, and other roles [10].

Functional decline in hospitalized elderly patients similarly refers to the decrement in a patient’s physical and/or cognitive functioning mostly reflected in his or her deterioration in self-care ability during hospitalization [11]. Factors associated with functional decline in hospitalized elderly patients include advanced age, impaired physical mobility, cognitive impairment, fall, malnutrition, and socioeconomic status [12,13,14].

The most frequently used instruments for predicting functional status among hospitalized elderly patients include the Identification of Seniors at Risk (ISAR) [15], Triage Risk Screening Tool (TRST) [16], Variables Indicative of Placement Risk (VIP) [17], Identification of Seniors at Risk—Hospitalized Patients (ISAR-HP) [18], Hospital Admission Risk Profile (HARP) [19], Care Complexity Prediction Instrument (COMPRI) [20], and Score Hospitalier d’Evaluation du Risque de Perte d’ Autonomie (SHERPA) [21]. The ISAR and TRST are primarily tested in the emergency department, with functional decline used as the primary outcome [15,16]. The VIP, previously tested for predicting discharge problems and increased length of stay, has been used in both the emergency department and wards [17]. The ISAR-HP, HARP, COMPRI, and SHERPA have been mainly tested in the wards, and used in nursing home institutionalization, with functional decline as their primary outcomes [18,19,20,21]. However, studies comparing the sensitivity, specificity, and optimal cutoff points of the aforementioned screening instruments have still published inconsistent results [22,23]. 

For the purposes of this study, we chose three specific tools for predicting functional status among hospitalized elderly patients. The ISAR-HP [18] and VIP [17] have only four items that can be easily, quickly, and widely used in clinical practice, with the scope of measurement covering both ADLs and IADLs. The SHERAP [21] has 32 items that contain the 21-item Mini-Mental State Examination (MMSE), as it is important to identify cognitive impairment as a risk factor for functional decline. Nevertheless, there has been a lack of comparative research determining whether the number of the items and scope of measurement affects the accuracy of prediction. Therefore, the current study aimed to evaluate and compare the diagnostic accuracy of the ISAR-HP, VIP, and SHERPA in predicting functional decline 30 days after discharge in older patients admitted to an acute hospital ward.

## 2. Methods

### 2.1. Study Design and Participants

This prospective, longitudinal study used a purposive sampling method to enroll inpatients aged 65 and over at the internal medicine ward of a teaching hospital in central Taiwan. The inclusion criteria included clear consciousness, no history of dementia and mental disorder, and no speech impairment. The exclusion criteria were disturbances in consciousness, dementia, cancer or terminal illness, hospitalization for less than 48 h, critical condition, and total dependence. 

### 2.2. Ethical Considerations

This study was approved by the responsible institutional review board (No.: P10510), and written consent had been obtained from all enrolled subjects before initiating data collection.

### 2.3. Data Collection

Data were collected from 2 May 2016 to 22 February 2017 using the first assessment conducted within 48 h of hospitalization. All baseline data were collected by a trained research nurse who interviewed the patients. Baseline data included the three screening instruments (ISAR-HP, VIP, and SHERPA), age, sex, polypharmacy (i.e., ≥5 types), premorbid functional status (i.e., 2 weeks before hospitalization), and length of stay. The second assessment was performed through a telephone interview 30 days after hospital discharge.

**Figure 1:**
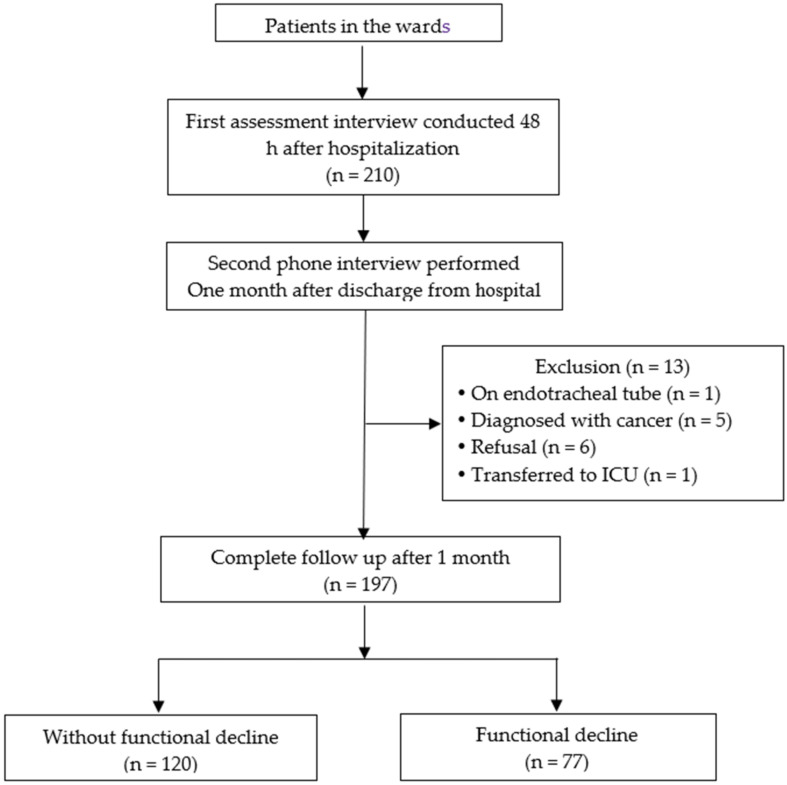
Flow chart of patient selection.

Among the 210 enrolled subjects, one was intubated and unable to communicate, five were diagnosed with cancer, six declined the telephone follow-up, and one was transferred to the intensive care unit due to critical condition, resulting in the withdrawal of 13 subjects (6.2%) from the study. A total of 197 subjects completed the study (Figure 1).

### 2.4. Measurement Instruments

#### 2.4.1. Functional Status

The Barthel Index [24], a useful standardized scale to assess functional disability, includes ten personal activities, namely feeding, moving between the wheelchair and bed, personal toileting, getting on and off a toilet, bathing, walking on a level surface (or propelling a wheelchair if unable to walk), ascending and descending stairs, dressing and undressing, controlling bowel, and controlling bladder. Basic care items (bathing and grooming) are scored as 0 (dependent) or 5 (independent), whereas more complex care needs (e.g., walking and transferring) were scored as 0 (dependent), 5 (major help), 10 (minor help), or 15 (independent). The Barthel Index scores were transformed to 0–100 scaling, with higher scores indicating better levels of independence. The Barthel Index has been shown to have good psychometric properties [25,26]. Patients were asked to rate their ADL function 2 weeks before admission to reduce the impact of the post-hospital illness on functional status [27]. For the purpose of this study, the prompting admission functional decline was defined as a decline of at least 5 points in the Barthel Index 30 days after discharge compared to pre-admission scores [22].

#### 2.4.2. Three Screening Instruments

The ISAR-HP [18] is a screening instrument used to predict the 90-day functional decline in acutely ill older patients admitted to the internal medicine ward. The ISAR-HP is a score card with four yes/no questions on the need for regular assistance with IADLs, use of a walking device, the need for assistance with traveling, and continued education after 14 years of age. Scores can range from 0 to 5, with a score of ≥2 indicating risk for functional decline. The psychometric properties of the ISAR-HP were acceptable for older patients acutely admitted and hospitalized for ≥48 h, with a sensitivity of 87%, a specificity of 39%, and an area under the curve (AUC) of 0.71 [18].

The VIP [17] assesses older patients’ degree of independence and was developed to identify hospitalized patients aged ≥70 years at risk of post-discharge problems. For 4-item combinations, a patient is considered to be at increased risk of discharge problems when ≥3 items are reported positive using yes/no questions. The psychometric properties of the VIP were acceptable for hospitalized older patients. Although the sensitivity of VIP was too low (62%) at a cutoff ≥2, this could be optimized with a cutoff score of ≥1, which demonstrated a sensitivity, specificity, positive predictive value, and negative predictive value of 88%, 21%, 48%, and 68%, respectively [22]. Moreover, at a cutoff score of <2, the VIP indicated good accuracy in predicting discharge problems and extended length of stay [22].

The SHERPA is a predictive tool developed by Cornette et al. [21] in France for the identification, upon hospital admission, of elderly patients at risk of functional decline 3 months after discharge. SHERPA contains five categories: fall within the previous year, short version (21-point) MMSE, bad self-perceived health, age, and pre-admission IADL score. This tool categorizes risk into four levels: low risk (0–3), mild risk (<3.5), moderate risk (<5), and high risk (≤6). With a cutoff at 4, the sensitivity, specificity, and AUC of the SHERPA were estimated to be 67.9%, 70.8%, and 0.73, respectively.

### 2.5. Statistical Analyses

The three instruments for screening functional decline, namely ISAR-HP, VIP, and SHERPA, were analyzed in terms of sensitivity, specificity, and receiver operating characteristic (ROC) curve. Moreover, Youden’s Index was adopted to identify the best cutoff for each instrument. The index both measures the effectiveness of a diagnostic tool and facilitates the selection of an optimal cutoff point for the tool by estimating the sensitivity and specificity of every cutoff on the ROC curve based on the formula: Sensitivity + Specificity − 1. The estimated value should fall between 0 and 1, with scores closer to 1 indicating a better trade-off between sensitivity and specificity [28].

## 3. Results

This study included 93 male and 104 female subjects with a mean age of 77.7 years. Most of the subjects were aged between 75 and 84 years (45.6%), with 49.2% receiving only elementary education, 73.6% being married, 68% living with their families, and 72.5% being hospitalized after an emergency room visit. Approximately 43.6% reported taking more than five medications, 55.3% had a MMSE score of ≤14 points, and 60.9% perceived poor health. The length of hospital stay was 6.5 days (Table 1).

Among the 197 subjects, 39.1% experienced functional decline, whereas the remaining 60.9% showed no sign of functional decline. The average of the first and the second Barthel Index scores of the groups with functional decline were 81.88 ± 20.80 48 h after hospitalization and 69.48 ± 21.14 30 days after discharge, respectively. Significant differences in age (*p* = 0.021), short version (21-point) MMSE scores (*p* < 0.001), and perception of poor health (*p* < 0.001) were observed between both groups (Table 1).

**Figure 2:**
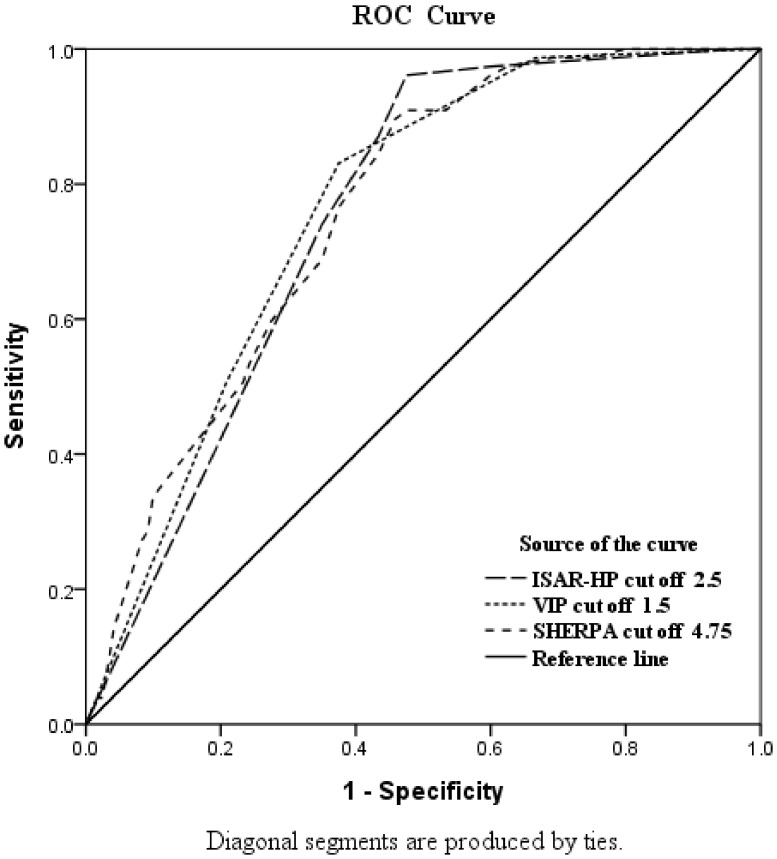
Area under the receiver operating characteristic for ISAR-HP, VIP, and SHERPA.

As Figure 2 and Table 2 show, the ROC curve analyses revealed that the AUC of ISAR-HP was 0.751 (95% CI: 0.684–0.818). According to Youden’s Index, the best cutoff was estimated to be 2.5 points, at which the sensitivity and specificity were 96.1% and 52.5%, respectively. For VIP, the AUC was 0.761 (95% CI: 0.695–0.826), and the best cutoff was 1.5 points, at which the sensitivity and specificity were 83.1% and 62.5%, respectively. For SHERPA, the AUC of SHERPA was 0.758 (95% CI: 0.692–0.823) and the best cutoff was estimated to be 4.75 points, at which the sensitivity and specificity were 89.6% and 54.2%, respectively. The ISAR-HP emerged to outperform SHERPA and VIP in terms of predictive power.

## 4. Discussion

The present study has been the first to verify whether VIP, ISAR-HP, and SHERPA can be used to predict functional decline 30 days after hospital discharge among elderly people over 65 in Taiwan. Our results showed that 39% of the subjects exhibited functional decline 30 days after discharge, consistent with the results of Deschodt et al. (2011) [22]. In contrast, Zisberg et al. (2015)’s study [29] showed that 46.3% of the patients (317/684) exhibited a functional decline, with this difference perhaps being attributed to the inclusion of patients aged 70 and older, unlike our study which included those over 65.

However, Sager et al. [3] and Cornette et al. [21] found a 30–31.5% incidence rate of functional decline 3 months after discharge in hospitalized older patients, and Brase et al. [23] demonstrated that 22.6% of patients experienced functional decline. The aforementioned studies showed that the proportion of functional decline 30 days after discharge may be higher than that at 3 months after discharge. Functional changes among the hospitalized elderly is a dynamic process that may continue to occur from admission to discharge [29,30,31]. Therefore, it is very important to identify a tool that can predict functional decline within 30 days of discharge for early intervention programs to reverse the progression of functional decline. Furthermore, compared to those without functional decline, our study showed that subjects with functional decline were very elderly and had a lower MMSE score, supporting the findings of previous studies suggesting that advanced age and cognitive impairment were two major risk factors of functional decline [14,32,33].

Past studies have shown that the adjustment of cutoff scores can affect the sensitivity and specificity of the tool [34]. Therefore, our study used Youden’s Index analysis to identify the best cutoff score. We found that ISAR-HP, VIP, and SHERPA demonstrated an AUC falling in the range of 0.75–0.76, indicating moderate diagnostic accuracy [35]. Moreover, ISAR-HP had a better sensitivity than VIP and SHERPA.

This study has been the first to utilize the ISAR-HP and SHERPA as a tool for predicting functional decline 30 days after discharge. Previously, the ISAR-HP [18,34,36] and SHERPA [21,37] had been used to predict functional decline after 3 months. However, our analysis of the ISAR-HP using Youden’s Index [28] showed that when the best cutoff point was 2.5 (up 0.5 from the original value), the AUC, sensitivity, and specificity were 0.751, 96.1, and 52.5, respectively, which were better than Hoogerduijn et al.’s [18]. Given that the four items of the ISAR-HP include ADL and IADL, the sensitivity and specificity should also increase together with these scores. As suggested by de Gelder et al. (2017) [34], increasing the cutoff point can help increase the positive predictive value. As for the SHERPA, our analysis using Youden’s Index [28] showed that when the best cutoff point was 4.75, the AUC, sensitivity, and specificity were 0.758, 89.6%, and 54.2%, which were better than the original work by Cornette et al. [21]. Our cutoff score (4.75) was equivalent to moderate risk in the findings of Cornette et al. [21], although our sensitivity (89.6%) was better than that of the original work (67.9%) [21]. The five factors of the SHERPA scale, including age, impairment in premorbid IADLs, falls within the years before hospitalization, cognitive impairment, and poor self-rated health, were related to functional decline [14]; thus, a moderate risk on this scale can predict functional decline 30 days after discharge.

Although the original purpose for the development of the VIP was to identify the elderly at risk of discharge problems from the hospital [17], some studies have also used it to predict the risk of functional decline within 3 months of discharge [23]. Deschdos et al. (2011) predicted functional decline within 1 month after discharge [22] and found that the cutoff score needs to be lowered to increase sensitivity. Our study used Youden’s Index and found that function is predicted to decline 30 days after discharge, and that the best cutoff point score of 1.5 yielded a sensitivity, specificity, and AUC of 83.1%, 62.5%, and 0.761, respectively. These findings suggested that the low risk category of the scale can predict functional decline 30 days after discharge.

The current study showed that all three instruments were capable of screening functional decline in elderly patients 30 days after hospital discharge. At the optimal cutoff values estimated herein, ISAR-HP and SHERPA, in particular, demonstrated better sensitivity and specificity than those reported in the original studies.

## 5. Conclusions

The present study found that ISAR-HP, VIP, and SHERPA were all capable of screening functional decline among hospitalized elderly patients, and should therefore be used as effective screening tools to facilitate the early detection of functional decline and to prevent its irreversible outcomes. Because ISAR-HP, VIP and SHERPA are effective, efficient and easy to use, they can be introduced into clinical settings for predicting the functional decline of hospitalized elderly patients. The timely use of these screening tools can effectively prevent and delay functional decline, thereby reducing disability and health care costs.

## 6. Limitations

The current study collected post-discharge data via telephone follow-up, an approach that is more susceptible to unexpected interferences. A face-to-face interview, although more costly and time-consuming, can better safeguard against interferences, and is thus believed to be a better approach for collecting data 30 days after hospital discharge in future research. Moreover, this study focused exclusively on patients at the internal medicine ward, which may decrease the generalizability of the current findings. Future research should therefore consider increasing the diversity of hospitalized patients to improve the generalizability of the results.

## Figures and Tables

**Table 1:** Demographic and clinical characteristics of patients with and without functional decline.

Variables	All (N = 197)		Without Functional Decline (n = 120)		Functional Decline (n = 77)		*p*
	n (%)	M ± SD	n (%)	M ± SD	n (%)	M ± SD	
Gender							0.849 ^b^
Males	93 (47.2)		56 (46.6)		37 (48.0)		
Females	104 (52.7)		64 (53.4)		40 (52.0)		
Age (years)		77.7		76.52 ± 7.19		78.9 ± 6.85	0.021 ^a,^*
65–74	73 (37.0)		51 (42.5)		22 (28.5)		0.132 ^b^
75–84	90 (45.6)		51 (42.5)		39 (50.7)		
≥85	34 (17.2)		18 (15.0)		16 (20.8)		
Education level							0.562 ^b^
Illiterate	75 (38.0)		41 (34.1)		34 (44.2)		
Elementary school	97 (49.2)		62 (51.6)		35 (45.4)		
Junior high school and above	25 (12.6)		17 (14.1)		8 (10.4)		
Marital status							0.287 ^b^
Single	5 (2.5)		5 (4.1)		0 (0.0)		
Married	145 (73.6)		88 (73.3)		57 (74.1)		
Widowed	42 (21.3)		25 (20.8)		17 (22.1)		
Divorced	5 (2.5)		2 (1.6)		3 (3.8)		
Caregiver							0.102 ^b^
Him or herself	24 (12.1)		20 (16.6)		4 (5.2)		
Family members	134 (68.0)		79 (65.8)		55 (71.4)		
Nurse aides	39 (19.7)		21 (17.5)		18 (23.4)		
Admitted from							0.770 ^b^
ER	143 (72.5)		88 (73.3)		55 (71.4)		
OPD	54 (27.4)		32 (26.7)		22 (28.6)		
Polypharmacy ≥5							0.060 ^b^
No	111 (56.3)		74 (61.6)		37 (48.1)		
Yes	86 (43.6)		46 (38.4)		40 (51.9)		
Barthel Index							
First assessment		83.5		85.29 ± 23.0		81.88 ± 20.80	0.284 ^a^
Second assessment		77.4		85.29 ± 23.0		69.48 ± 21.14	<0.001 ^a,^***
MMSE ^1^				14.33 ± 3.35		12.77 ± 3.02	<0.001 ^a,^***
0–14 (impaired cognition)	109 (55.3)		55 (45.8)		54 (70.1)		<0.001 ^b,^***
≥15 (normal cognition)	88 (44.6)		65 (54.2)		23 (29.9)		
Perception of poor health							
No	77 (39.0)		68 (56.7)		9 (11.7)		<0.001 ^b,^***
Yes	120 (60.9)		52 (43.3)		68 (88.3)		
Length of hospital stay (days)		6.5		6.39 ± 4.99		6.6 ± 3.27	0.727 ^a^

Note. ^1^ MMSE = Mini-Mental State Examination; * *p* < 0.05. *** *p* < 0.001; ^a^ Independent sample t test. ^b^ Chi-square test.

**Table 2:** The predictive values of three screening instruments predicting functional decline in older hospitalized patients.

	Original Cut-Off Points	Sensitivity (%)	Specificity (%)	Youden’s Index	The Best Cutoff Point	AUC (95% CI)
ISAR-HP ^1^	Total Score: 5 ≥2: risk	96.1	52.5	0.486	2.5	0.751 (0.684–0.818)
VIP ^2^	Total Score: 4 0–2: low risk 3–4: increased risk	83.1	62.5	0.456	1.5	0.761 (0.695–0.826)
SHERPA ^3^	Total Score 11.5 0–3: low risk 3.5–4.5: mild risk 5–6: moderate risk >6: high risk	89.6	54.2	0.438	4.75	0.758 (0.692–0.823)

Note. ^1^ ISAR-HP = the Identification of Seniors at Risk-Hospitalized Patients; ^2^ VIP = Variable Indicative of Placement Risk; ^3^ SHERPA = Score Hospitalier d’ Evaluation du Risque de Perte d’Autonomie.

## Data Availability

The data presented in this study are available on request from the corresponding author. The data are not publicly available due to privacy.
